# Trapezoid Fracture Associated with Scaphoid Fracture in a Football Goalkeeper

**DOI:** 10.1155/2019/7949754

**Published:** 2019-09-05

**Authors:** Tetsuya Yamamoto, Takehiko Matsushita, Kenjiro Ito, Shinji Matsushima, Kazuya Yoshida, Ryosuke Kuroda

**Affiliations:** ^1^Department of Orthopaedic Surgery, Kobe University Graduate School of Medicine, Kobe, Hyogo, Japan; ^2^Department of Orthopaedic Surgery, Akashi Medical Center, Akashi, Hyogo, Japan; ^3^Department of Orthopaedic Surgery, Aijinkai Rehabilitation Hospital, Takatsuki, Osaka, Japan

## Abstract

**Introduction:**

Trapezoid fractures are uncommon in sports. We presented a rare case of a trapezoid fracture associated with a scaphoid fracture caused by punching a ball in a football goalkeeper.

**Case Presentation:**

A 19-year-old male who played as a football goalkeeper visited our hospital with complaints of sustained pain from the right wrist to the hand after punching a ball. Scaphoid fracture was diagnosed on plain radiographs, whereas trapezoid fracture was overlooked. Computed tomography revealed a displaced trapezoid fracture associated with a scaphoid fracture. Both fractures were successfully treated by open reduction and internal fixation using cannulated screws. Almost complete bone union was achieved at 5 months after surgery. The patient returned to play as a football goalkeeper.

**Conclusion:**

The simultaneous occurrence of trapezoid and scaphoid fractures has never been reported. Trapezoid fractures are rare and can be overlooked on plain radiographs, as what happened in the present case, because the trapezoid is small and overlaps with other carpal bones on plain radiographs. If there is sustained pain in the wrist and hand after punching, combined trapezoid and scaphoid fractures should be considered as the possible injury.

## 1. Introduction

Scaphoid fractures are often observed after trauma, whereas trapezoid fractures are relatively rare, with only scattered reports in the literature [1–3]. A previous report indicated that scaphoid and trapezoid fractures accounted for 68.2% and 0.4% of all carpal bone fractures, respectively [4]. With respect to the mechanism of injury, there are some reports on scaphoid fractures due to punching [5, 6]. However, the simultaneous occurrence of trapezoid and scaphoid fractures in sports has never been reported. We presented a rare case of a trapezoid fracture associated with a scaphoid fracture caused by punching a ball in a football goalkeeper.

## 2. Patient Information/Clinical Findings

A 19-year-old male who played as a football goalkeeper visited our hospital with complaints of sustained pain from the right wrist to the hand after punching a ball. In the emergency department, the patient exhibited swelling at the right wrist and tenderness at the anatomical snuffbox of the right wrist and second metacarpal base area.

## 3. Diagnostic Assessment

**Figure 1:**
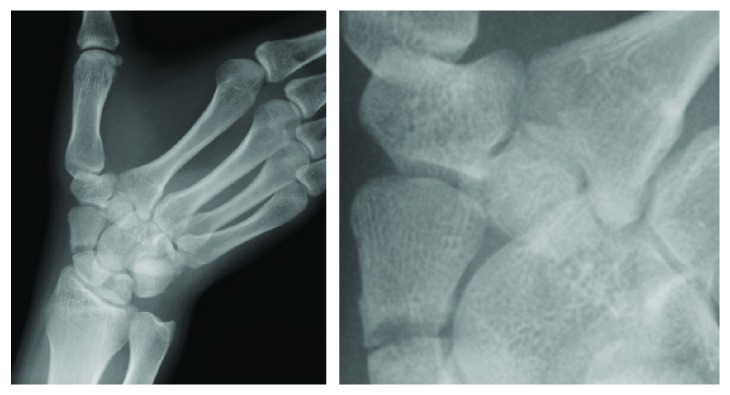
X-ray films showing displaced fracture of the trapezium and no other fractures.

**Figure 2:**
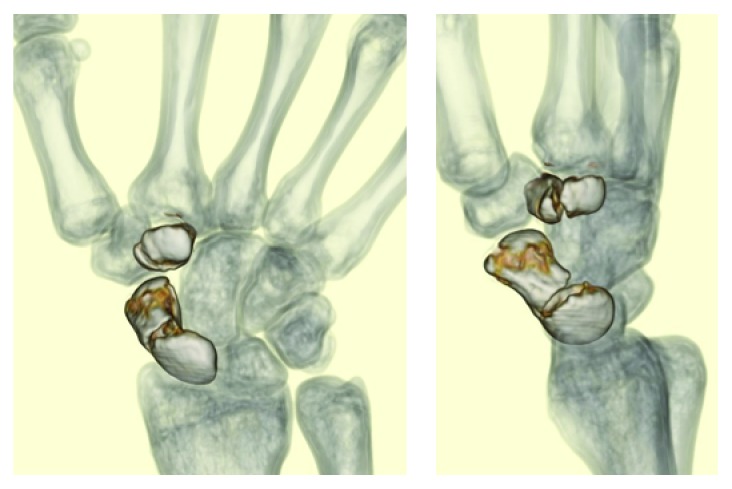
CT scan demonstrating fractures of the trapezium and the scaphoid.

Plain radiographs of the right wrist showed a displaced scaphoid body fracture (type 2B according to the Herbert classification). No other fractures could be identified on plain radiographs (Figure 1). Computed tomography (CT) scan was performed for the right wrist to obtain details on the fractured scaphoid. In addition to the displaced scaphoid body fracture, the CT scan revealed a displaced trapezoid fracture that was not clearly observed on plain radiographs. The fracture pattern of the trapezoid was vertical (Figure 2).

## 4. Therapeutic Interventions and Follow-Up

**Figure 3:**
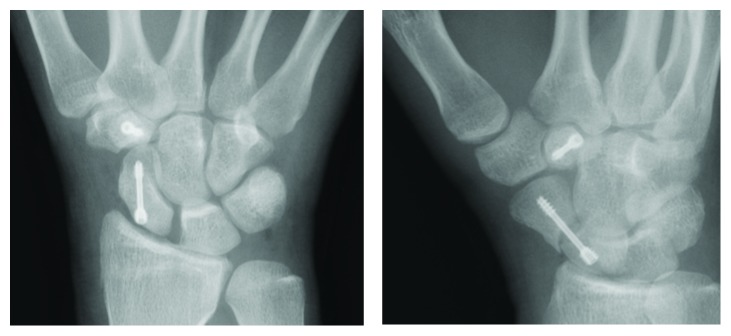
Postoperative X-ray films showing internal fixation with headless bone screws.

**Figure 4:**
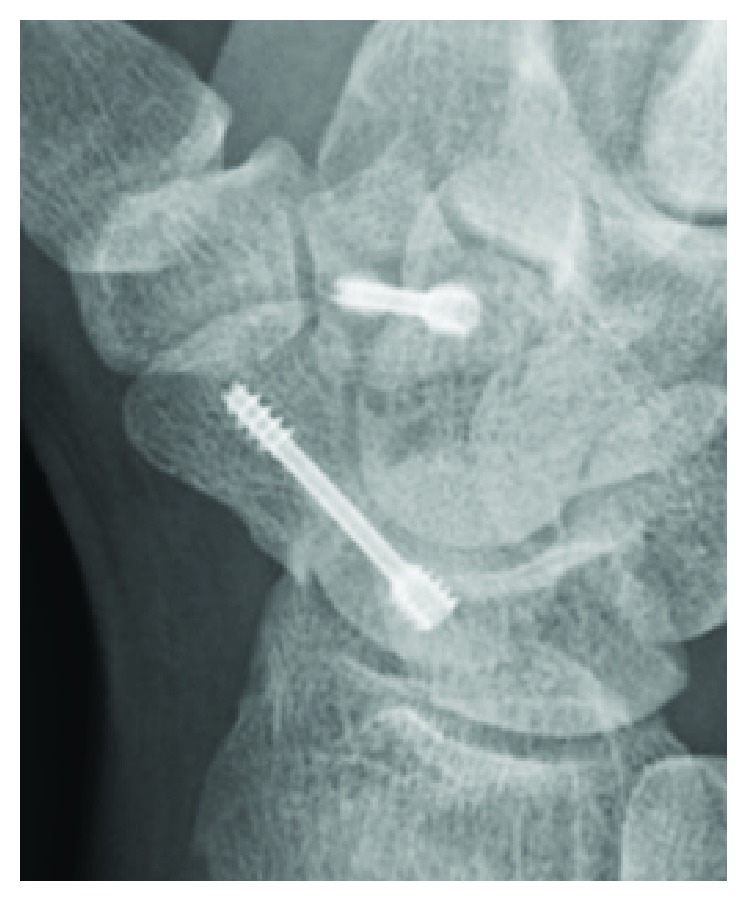
X-ray films at the 5-month follow-up visit. The fracture line was still visible in the scaphoid, but most part of the fracture site was united.

Surgery was performed at 11 days after the patient's initial hospital visit. Open reduction and internal fixation of the fractures was performed with two 1 cm minimal incisions over the scaphoid and trapezoid using a cannulated headless compression screw (double-threaded screw; MEIRA Corporation, Nagoya, Japan) for each fracture (Figure 3). After surgery, the patient's right wrist was placed in a short-arm thumb spica cast for 2 weeks. Range of motion exercise was initiated at 2 weeks after surgery. The fracture line was still visible in the scaphoid, but most part of the fracture site was united at 5 months after surgery (Figure 4). The patient returned to play as a football goalkeeper and is currently free of symptoms.

## 5. Discussion

The most notable finding was the occurrence of a trapezoid fracture associated with a scaphoid fracture caused by punching a ball in a football goalkeeper. Acute injuries, including finger fractures and thigh muscle injuries, have been reported to occur more frequently than overuse injuries in adolescent football goalkeepers [7]. Scaphoid fractures are often observed in sports activities; in contrast, trapezoid fractures are uncommon. To the best of our knowledge, the simultaneous occurrence of trapezoid and scaphoid fractures has never been reported in the English literature. Scaphoid fractures usually occur when the wrist is hyperextended during injury, although other mechanisms have also been suggested. Horii et al. examined scaphoid fractures resulting from punching and reported that the wrist position while punching was neutral to slight palmar flexion. Furthermore, they postulated that the axial load to the second metacarpal bone was transmitted to the scaphoid and subsequently produced a shearing force to the scaphoid waist, resulting in fractures [8]. In the present case, the patient had swelling and pain around the second metacarpal base of the right hand. Anatomically, the trapezoid is located among the metacarpal bones, trapezium, capitate, and scaphoid, and its movement is constrained by surrounding ligaments [9]. Therefore, it is possible that the axial load to the second metacarpal bone by punching was transmitted to the scaphoid via the trapezoid without being dispersed, leading to the fractures.

In the present case, the trapezoid fracture was not diagnosed on plain radiographs and overlooked. Diagnosing trapezoid fracture on plain radiographs is generally difficult, as the trapezoid is small and overlaps with other carpal bones. It has been reported that less than half of cases were improperly diagnosed at initial hospital visit, with the trapezoid fracture in one patient having been identified only after 99 days [10]. Together with our case report, this report suggests that trapezoid fractures can be easily overlooked on routine plain radiographs, especially in cases associated with other fractures. Therefore, if the patient complains of pain in the hand and scaphoid fracture is observed on plain radiographs, it is recommended that a CT scan be performed to prevent misdiagnosis.

Considering the rarity of trapezoid fractures, no established treatment method for them currently exists. Jeong et al. reported that a ≤2 mm displacement can be conservatively treated [11]. Some reports recommended that surgical treatment be selected depending on articular surface displacement and that open reduction and internal fixation be performed to restore articular congruity [12, 13]. In the present case, surgery was performed as treatment for trapezoid and scaphoid fractures owing to the estimated 3 mm displacement of the trapezoid fracture. Scaphoid fractures are often surgically treated in highly active patients. Therefore, it may be advisable to surgically treat trapezoid fractures if they are associated with scaphoid fractures to prevent displacement and nonunion.

## 6. Conclusion

We presented a rare case of a trapezoid fracture associated with a scaphoid fracture caused by punching a ball in a football goalkeeper. A trapezoid is constrained and received external force without being dispersed, so it is possible that the axial load to the second metacarpal bone by punching was transmitted to the scaphoid via the trapezoid, leading to the fractures. If there is sustained pain in the wrist and hand after punching, combined trapezoid and scaphoid fractures should be considered as the possible injury.
